# Scalable nanofocusing heterojunctions for raptor-inspired on-chip vision perception

**DOI:** 10.1126/sciadv.aee7840

**Published:** 2026-07-23

**Authors:** Lanhao Qin, Jiazheng Wang, Gaohang Huo, Shenghong Liu, Ming Deng, Jialiang Wang, Decai Ouyang, Wenke He, Lizhou Miao, Yi Xu, Hongbo He, Yajie Wang, Haohui Peng, Wenhua Hu, Yuan Li, Yang Chai, Tianyou Zhai

**Affiliations:** ^1^ State Key Laboratory of New Textile Materials and Advanced Processing, School of Materials Science and Engineering, Huazhong University of Science and Technology, Wuhan 430074, China.; ^2^ State Key Laboratory of Materials Processing and Die & Mould Technology, School of Materials Science and Engineering, Huazhong University of Science and Technology, Wuhan 430074, China.; ^3^ Department of Applied Physics, The Hong Kong Polytechnic University, Hong Kong, 999077, China.; ^4^ Dependable and Secure Computing Laboratory, School of Computer Science and Artificial Intelligence, Wuhan University of Technology, Wuhan 430070, China.; ^5^ Research Institute of Huazhong University of Science and Technology in Shenzhen, Shenzhen 518063, China.

## Abstract

Biological visual systems provide a blueprint for intelligent perception in complex dynamic environments. Inspired by the optical role of retinal oil droplets in raptor vision, we report a plasmonic nanofocusing synaptic array (PNSA) for bioinspired visual sensing and motion perception. The pivotal design is a wafer-scale Au@MoS_2_ core-shell heterojunction that enhances light concentration like the retinal oil droplets of raptors. Leveraging strong localized surface plasmon resonance in the Au core and efficient hot-carrier generation in the MoS_2_ shell, the PNSA exhibits an ultrafast relaxation time of 2.2 ms, corresponding to a theoretical refresh rate of ∼450 Hz. Uniform 2-inch wafer-scale fabrication enables a 32 × 32 optoelectronic synaptic array with 100% yield and only 2.2% device-to-device variation. The array supports 5-bit optical programming and generates temporally encoded motion representations that achieve 98.95% accuracy in motion-direction classification after 10 training epochs. This work establishes a scalable materials platform for bioinspired visual sensing, offering a promising route toward on-chip vision perception.

## Teaser

An nanofocusing synaptic array for fast visual intelligence is reported.

## INTRODUCTION

Accurate acquisition and continuous tracking of high-speed moving objects in complex dynamic environments remain critical challenges in applications such as autonomous driving, intelligent traffic systems, and surveillance (*1*–*3*). These scenarios require not only high sensitivity to light intensity and image features but also rapid temporal perception and reliable information storage (*4*–*8*). However, existing artificial image sensing technologies are still largely constrained by frame-based architectures, where sensing and processing are spatially and functionally separated (*9*–*12*), leading to high latency and reduced accuracy in real-time decision-making (*13*–*15*).

**Fig. 1.**
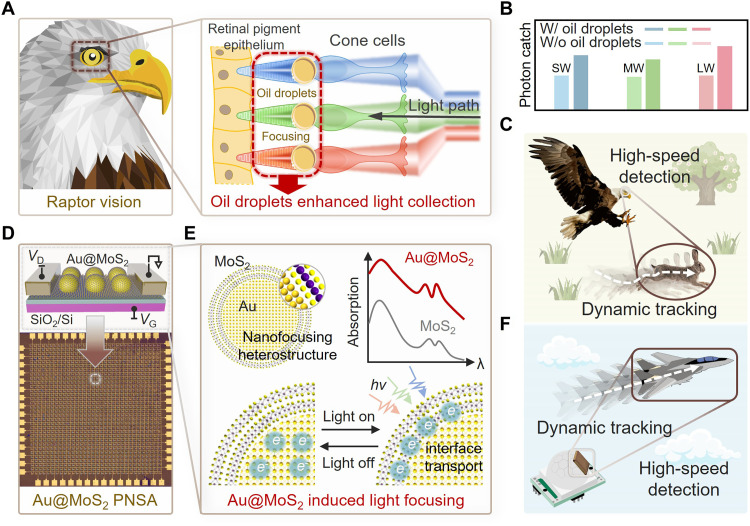
Raptor vision-inspired plasmonic nanofocusing synaptic array (PNSA) enables high-speed, on-chip visual detection and dynamic tracking. (**A**) Schematic illustration of raptor vision, highlighting oil droplets in retinal cone cells that enhance light collection. (**B**) Schematic comparison of photon capture capacity in retinal cells with and without oil droplets. (**C**) Ecological hunting scenario of raptors relying on acute visual perception. (**D**) OM image of Au@MoS_2_ arrays and rendering of a single device. (**E**) Mechanistic illustration of Au@MoS_2_-induced light nanofocusing, including the heterostructure’s light absorption spectrum and carrier transport behavior at the interface. (**F**) Application scenarios enabled by the PNSA, demonstrating high-speed detection and dynamic tracking of moving targets.

In contrast, raptors exhibit extraordinary visual capabilities, supported by specialized retinal architectures and efficient visual pathways (*16*–*18*). As illustrated in Fig. 1A, raptor retinas contain cone photoreceptors with retinal oil droplets positioned in front of the outer segments, which are proposed to facilitate light propagation and channel spectrally filtered light toward the outer segments, thereby enhancing photon capture in the outer segments (*19*, *20*). Figure 1B schematically illustrates this optical effect. Moreover, raptor visual signals are processed through a prominent tectofugal network centered on the optic tectum, which supports rapid visual processing, saliency selection, and motion-related computations in complex scenes (*21*–*23*). These optical and neural adaptations together provide a biological basis for the exceptional motion-sensitive vision of raptors, facilitating rapid target tracking during high-speed predation (Fig. 1C) and efficient processing of dynamic visual information (*24*–*27*).

Inspired by the optical role of retinal oil droplets and the rapid visual processing observed in avian systems, we propose a plasmonic nanofocusing synaptic array (PNSA) for bioinspired motion-sensitive visual sensing and dynamic perception at the hardware level (Fig. 1D). Central design to the PNSA is a wafer-scale Au@MoS_2_ core-shell heterojunction that provides enhanced light concentration inspired by the optical role of oil-droplet-containing cone cells in raptors. The Au nanoparticle core enables strong localized surface plasmon resonance (LSPR), substantially amplifying the near-field optical intensity and serves as an artificial analogue of the optical effect in biological oil droplets. Meanwhile, the MoS_2_ shell efficiently absorbs the concentrated light and generates hot carriers, enabling rapid photoelectric transduction for dynamic visual sensing (Fig. 1E) (*28*–*30*).

The heterojunctions are fabricated uniformly over a 2-inch wafer with >99.5% spatial uniformity, and the resultant 32 × 32 optoelectronic synaptic array achieves 100% yield and only 2.2% device-to-device variation. The PNSA demonstrates an ultrafast relaxation time of 2.2 ms, corresponding to a theoretical refresh rate of ∼450 Hz. Each synapse exhibits 5-bit optically programmable conductance states, ensuring high-resolution signal encoding. Using temporally encoded motion representations generated by the synaptic array, a convolutional neural network (CNN) achieves 98.95% direction recognition accuracy after only 10 training epochs, demonstrating the potential of this raptor-vision-inspired hardware framework for fast and precise visual perception and real-time in-sensor computing (Fig. 1F).

## RESULTS

### Scalable Au@MoS_2_ heterojunction synthesis

**Fig. 2.**
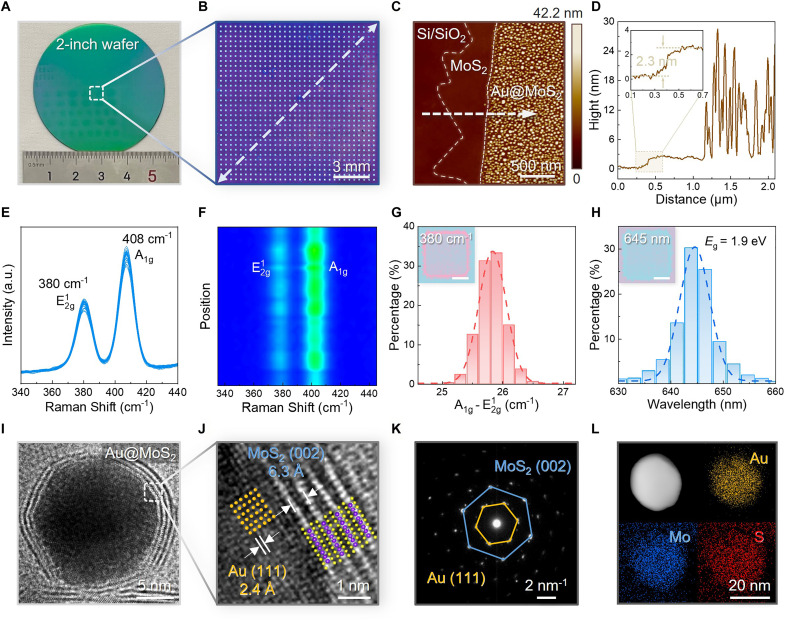
Fabrication and characterization of 2-inch wafer-scale Au@MoS_2_ heterojunctions. (**A**) Photograph of a 2-inch Au@MoS_2_ heterojunction grown on SiO_2_/Si substrate. (**B**) OM image of a 32 × 32 Au@MoS_2_ heterojunction array. (**C**) AFM image of Au@MoS_2_ heterojunction. (**D**) Line profile of the Au@MoS_2_ heterojunction, the inset is a local magnified height curve. (**E** and **F**) Raman spectrum (E) and Raman pseudocolor map (F) of 32 Au@MoS_2_ patterns in the diagonal area indicated by the dashed line in B. (**G** and **H**) Statistical distributions of Raman peak (G) and PL peak (H) positions of 32 Au@MoS_2_ patterns in the diagonal area indicated by the dashed line in (B). (**I** and **J**) TEM image of Au@MoS_2_ heterojunction (I) and its interface magnification image (J). (**K**) SAED pattern of Au@MoS_2_ heterojunction. (**L**) EDS elemental mapping of Au, Mo, and S elements.

The scalable synthesis of nanofocusing Au@MoS_2_ heterojunctions is realized via a seed-assisted chemical vapor deposition (CVD) strategy. A 2-inch wafer-scale heterojunction is shown in Fig. 2A, demonstrating the feasibility of large-area fabrication. A magnified view (Fig. 2B) reveals a well-ordered 32 × 32 array of heterojunctions, confirming the uniform distribution of Au@MoS_2_ across the wafer. Atomic force microscopy (AFM) characterization (Fig. 2C) illustrates the morphological contrast between the pristine MoS_2_ region (left) and the Au@MoS_2_ heterojunction region (right). The height profile along the dashed line (Fig. 2D) further quantifies the thickness. The inset shows a local MoS_2_ shell thickness of ∼2.3 nm on Au nanoparticles, while the overall heterojunction film reaches ∼14.2 nm. These results confirm the successful formation of Au@MoS_2_ core-shell structure accompanied by lateral epitaxial growth of planar MoS_2_ on the substrate. Figure S1A displays the Scanning electron microscopy (SEM) image of Au@MoS_2_ heterostructures, where the particles are uniformly dispersed across the substrate without apparent aggregation. The corresponding particle size distribution histogram (fig. S1B), reveals that the Au@MoS_2_ heterostructures exhibit an average particle size (μ) of 16.1 nm and a relatively narrow size distribution. X-ray photoelectron spectroscopy (XPS) and ultraviolet photoelectron spectroscopy (UPS) further confirm the chemical states and interfacial energy-band structure of the Au@MoS_2_ heterojunctions (figs. S2 and S3). Such uniform particle size and dispersion, enabled by the controllable synthesis strategy, are essential for achieving reproducible and homogeneous optoelectronic performance in Au@MoS_2_-based devices, as they mitigate property variations caused by structural inhomogeneities.

Figure S4A shows the Raman spectra of Au@MoS_2_ and pristine MoS_2_. For Au@MoS_2_, the characteristic Raman peaks are observed at 406.15 cm^−1^ and 380.24 cm^−1^, corresponding to the A_1g_ and $E_{2g}^{1}$ modes, respectively. Compared with pristine MoS_2_, the A_1g_ mode of Au@MoS_2_ exhibits a redshift, while the $E_{2g}^{1}$ mode shows a blueshift, which is consistent with previously reported observations (*31*). The photoluminescence (PL) spectrum of pristine MoS_2_ displays emission peaks at ∼1.82 eV and ∼1.92 eV. In contrast, Au@MoS_2_ shows a distinct shift in PL peak positions (fig. S4B), which can be attributed to the strain in the MoS_2_ shell and the LSPR effect caused by Au nanoparticles. The LSPR effect enhances photon absorption and promotes non-radiative relaxation, thereby reducing the PL intensity of the Au@MoS_2_ heterojunction (*32*). In addition, the devices exhibit high responsivity, external quantum efficiency, and detectivity over a broad range of illumination powers and wavelengths (fig. S5), further supporting their excellent optoelectronic response.

To evaluate large-area uniformity, Raman spectra were collected along the diagonal of the Au@MoS_2_ array (Fig. 2E), showing nearly identical spectral features. The Raman pseudocolor map (Fig. 2F) further illustrates that the Au@MoS_2_ heterojunction films maintain excellent consistency in layer thickness and structural uniformity across the entire array. Wafer-scale Raman mapping of a 2-inch Au@MoS_2_ film was then performed, and statistical analysis of the peak separation between the A_1g_ and $E_{2g}^{1}$ modes (Fig. 2G) yield an average value of 25.8 cm^−1^ with a minimal fluctuation range, indicating excellent structural homogeneity. Similarly, PL mapping (Fig. 2H) reveals that the average emission peak of the Au@MoS_2_ heterojunctions is 644.3 nm, confirming outstanding optical uniformity. Collectively, these results establish that Au@MoS_2_ heterojunction films achieve remarkable optical and structural uniformity across the entire 2-inch wafer. Such wafer-scale consistency is a critical prerequisite for the development of high-performance, large-area integrated optoelectronic devices, ensuring reproducible and reliable device operation.

High-resolution transmission electron microscopy (HRTEM) is employed to investigate the microstructure of Au@MoS_2_ heterojunctions. As shown in Fig. 2I, MoS_2_ uniformly coats Au nanoparticles, forming well-defined core-shell heterojunctions. The interfacial region is further resolved in Fig. 2J, where an atomically clean and sharp contact between Au and MoS_2_ is clearly observed. The measured interlayer spacing of MoS_2_ is ∼6.3 Å, corresponding to the (002) crystal plane, while Au nanoparticles exhibit an interplanar distance of ∼2.4 Å, characteristic of the (111) plane (*33*). The selected area electron diffraction (SAED) pattern (Fig. 2K) further confirms the crystalline nature of the Au@MoS_2_ heterojunctions. The diffraction spots can be indexed to the (002) plane of MoS_2_ and the (111) plane of Au, demonstrating the epitaxial relationship between the two components. Energy dispersive x-ray spectroscopy (EDS) mapping (Fig. 2L) shows uniform spatial distribution of Au, Mo, and S. Notably, the Mo and S signals extend beyond the Au domain, corroborating the formation of Au@MoS_2_ core-shell heterojunctions.

### Au@MoS_2_ PNSA fabrication and uniformity

**Fig. 3.**
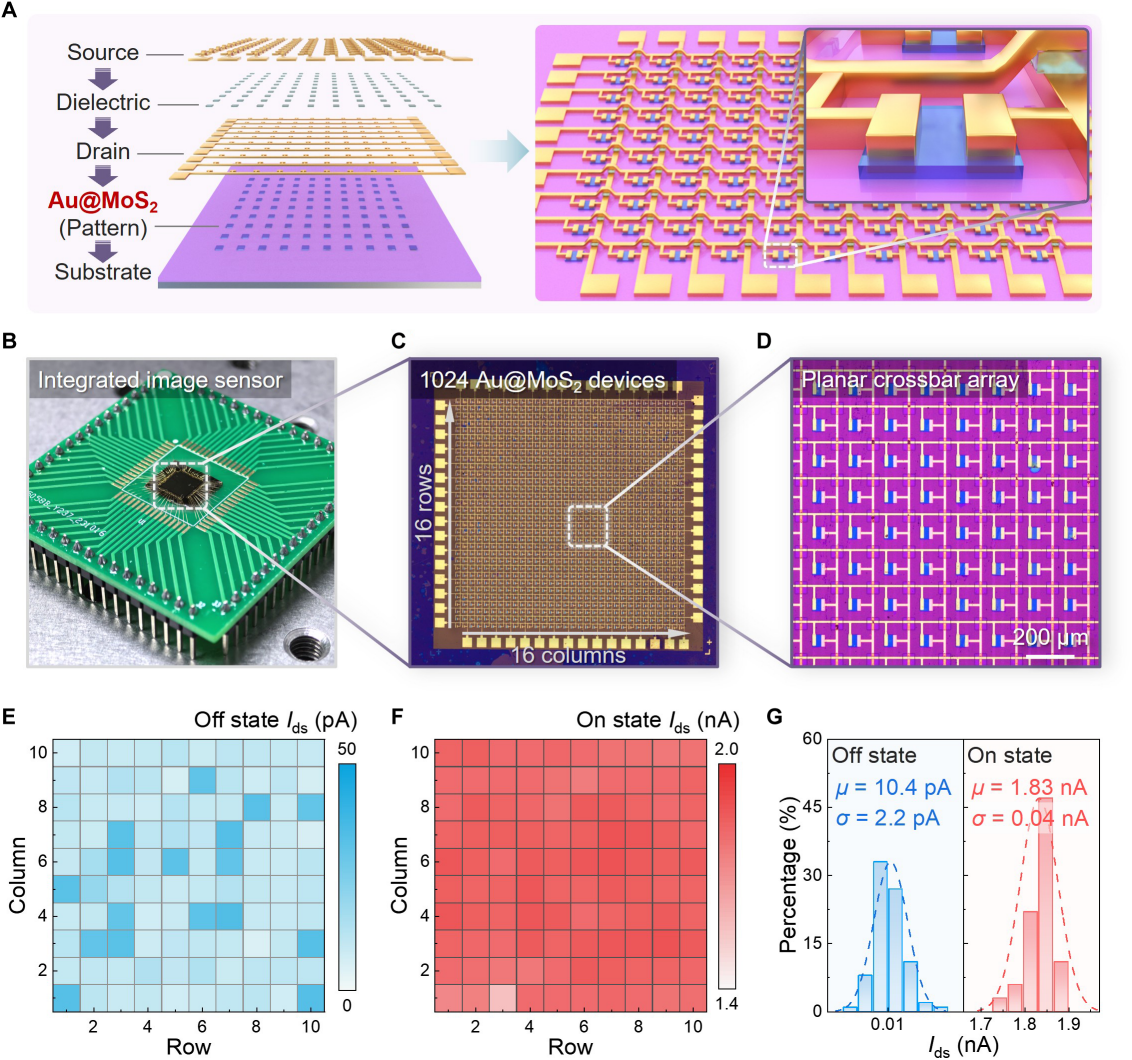
Integration and electrical uniformity of the Au@MoS_2_ PNSA. (**A**) Structure diagram and array rendering of the 32 × 32 Au@MoS_2_ PNSA. (**B**) Photograph of a wire-bonded Au@MoS_2_ PNSA. (**C**) OM image of the Au@MoS_2_ PNSA. (**D**) Locally enlarged OM diagram of the array device. (**E** and **F**) Mappings of off state (E) and on state (F) currents in an Au@MoS_2_ PNSA. (**G**) Distribution of off state and on state currents in an Au@MoS_2_ PNSA.

To further validate the practical viability of the Au@MoS_2_ heterojunctions for neuromorphic vision applications, we fabricated a 32 × 32 PNSA. The device architecture is schematically illustrated in Fig. 3A. A standard row-column addressing scheme is employed, wherein source and drain electrodes are patterned on opposing sides of each heterojunction element. A 100 nm-thick Si_3_N_4_ insulating layer is deposited at each electrode intersection to effectively suppress parasitic leakage currents and ensure electrical isolation across the array. Figure 3B presents the bounding integration of the Au@MoS_2_ PNSA into an image sensor platform. The optical microscopy (OM) image of the full 32 × 32 array (Fig. 3C) confirms the successful realization of high-density integration over a large area. A magnified view (Fig. 3D) clearly resolves the cross-point electrode structure and demonstrates the high patterning fidelity and reproducibility of the fabrication process.

To evaluate the electrical and photoresponse uniformity of the PNSA, a statistical analysis was conducted on 100 randomly selected devices under a drain bias (*V_ds_*) of 1 V and 532 nm laser illumination with an optical power density of 2.55 mW·cm^−2^. The drain current (*I_ds_*) was recorded before and after continuous laser exposure for 120 s. Figure 3, E and F, present the distribution of device currents in the dark (off state) and under illumination (on state), respectively. In the absence of light (Fig. 3E), the currents are tightly clustered at the pA level with minimal variation, indicating excellent baseline electrical uniformity across the array. Upon illumination (Fig. 3F), the currents increase substantially into the nA level, confirming a strong and consistent photoresponse. Quantitative analysis of these distributions is provided in Fig. 3G. The average off-state current is 10.4 pA with a standard deviation (σ) of 2.2 pA, while the average on-state current reaches 1.83 nA with σ = 0.04 nA. The resulting device-to-device variation rate is calculated to be only 2.21%, with an average on/off current ratio of 176. These values reflect outstanding device uniformity and robust photodetection characteristics across the array. The combination of 100% yield, tight variation margins, and sub-nanoampere noise levels underscores the high reproducibility and fabrication quality of the Au@MoS_2_ PNSA. This uniformity is critical for large-scale neuromorphic vision systems, as it enables accurate spatial and temporal signal processing across dense device networks without the need for complex individual calibration or compensation circuitry.

### Optoelectronic synaptic characteristics

To further elucidate the optoelectronic synaptic characteristics of Au@MoS_2_ devices, we measured their response to synergistic optical and electrical stimulations. As shown in fig. S6A, the device exhibits pronounced current modulation under three consecutive 532 nm light stimuli at *V*_ds_ = 1 V and *V*_G_ = 0 V. When a minimal gate voltage (*V*_G_ = 0.1 V) is applied, the current response is substantially enhanced, indicating that even a low gate voltage can effectively regulate carrier distribution in the device. This demonstrates that the Au@MoS_2_ device allows for flexible and adaptive control under synergistic optical and electrical stimulations. Figure S6B further presents the dependence of *I*_ds_ on the amplitude of the gate voltage pulse. A potentiation behavior (post-synaptic current potentiation, PPC) is observed at *V*_G_ = 10 V, while a depression behavior (post-synaptic current depression, NPC) is seen at *V*_G_ = 0 V. As the number of voltage pulses increases, both PPC and NPC effects become more pronounced, demonstrating the capability of the device to mimic synaptic plasticity through electrical pulse modulation.

Moreover, fig. S7A shows the *I*-*V* curves under dark conditions and varying 532 nm laser power densities (0.13–1.27 mW·cm^−2^). The photocurrent increases with light intensity, demonstrating a notable photoresponse. A power-law fitting between photocurrent (*I*_ph_) and incident light intensity (*P*_in_) yields a power exponent α ≈ 0.557 (fig. S7B). The sub-unity value of *α* suggests a prominent exciton recombination effect within the device, primarily attributed to interfacial defect states in the Au@MoS_2_ that facilitate carrier trapping (*34*, *35*). In addition, the paired-pulse facilitation (PPF) behavior of the device is characterized in fig. S7C. As the pulse interval increases from ∆*t*_0_ to 1000∆*t*_0_ (∆*t*_0_ = 25 ms), the PPF index decreases from ∼230% to ∼100%, indicating a strong facilitation response to short-interval stimulations. Fitting the decay yields two distinct relaxation time constants: a fast component of 2.03 s and a slow component of 219.44 s. This distinction suggests that the synaptic response involves both fast surface trapping/release and slow defect-state-mediated carrier dynamics, highlighting the crucial role of interfacial defect states in the synaptic behavior of the Au@MoS_2_ heterostructure.

Notably, under 20 Hz dual-pulse stimuli (fig. S7D), the fast relaxation time is reduced to only 2.2 ms, notably shorter than in conventional optoelectronic synaptic devices, demonstrating the potential of the Au@MoS_2_ device for high-speed optoelectronic applications. This ultrafast response, together with operating currents at the pA-to-nA range, translates into extremely low energy consumption per synaptic event—an essential attribute for large-scale neuromorphic systems in energy-constrained scenarios. The dynamic response under different light pulse frequencies (1 Hz, 10 Hz, and 100 Hz) is shown in fig. S7E. With increasing frequency, the rates of carrier injection and trapping per unit time increase, resulting in a transition from short-term plasticity (STP) to long-term plasticity (LTP). This is due to incomplete charge release between pulses (interval = 25 ms), leading to charge accumulation and the filling of more trap states, thus yielding a nonlinear current enhancement. This behavior is conducive to implementing synapse-like temporal integration functions.

### Optical encoding motion perception

**Fig. 4.**
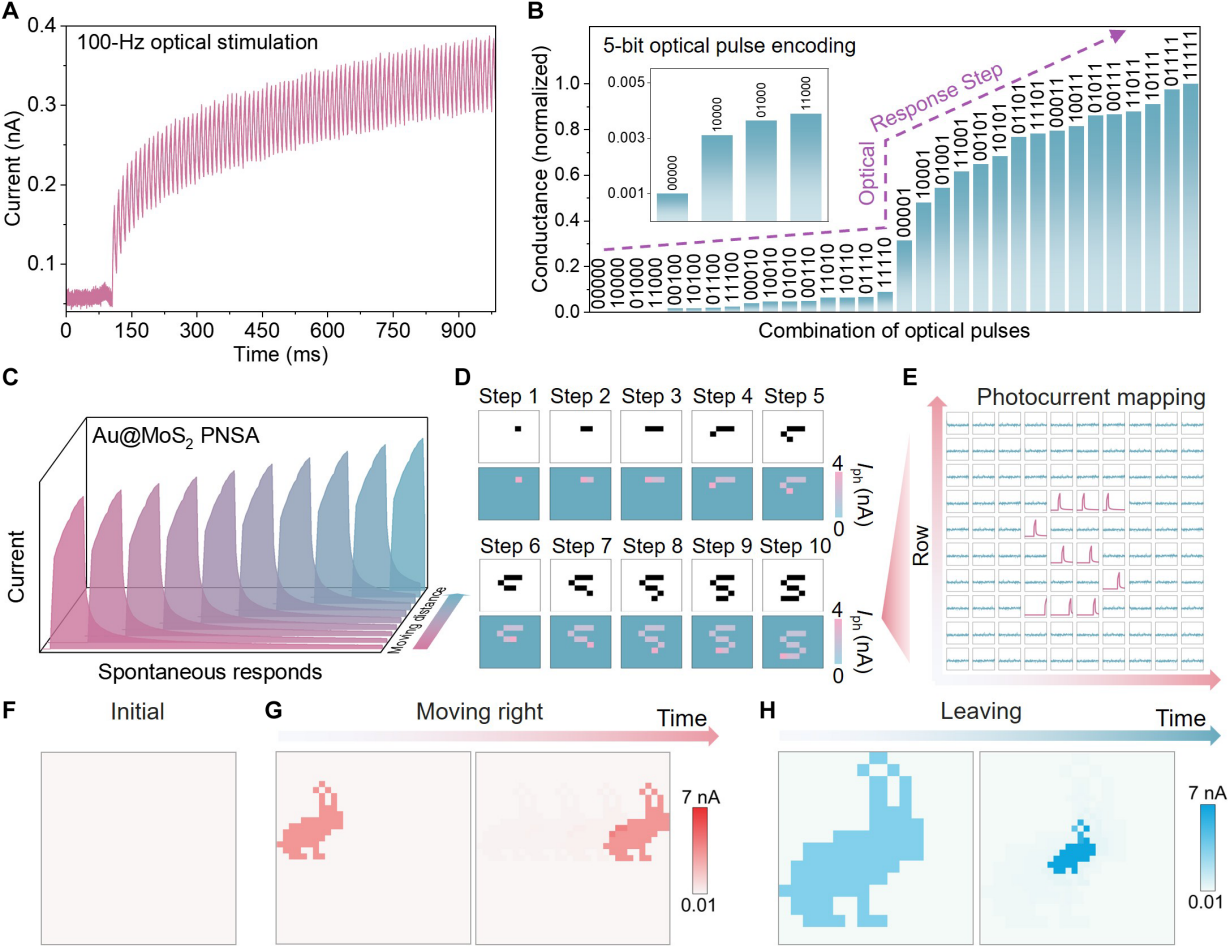
Optical encoding and dynamic tracking of the Au@MoS_2_ PNSA. (**A**) Photocurrent evolution under 100-Hz optical stimuli. (**B**) Stepwise response to 5-bit binary-coded optical pulse, where “1” and “0” denote light stimuli and dark, respectively. (**C** to **E**) In-sensor trajectory registration using the Au@MoS_2_ PNSA. Schematic illustration of motion detection based on spatially resolved photocurrent response (C); acquired trajectory images at each step of a moving light spot across the PNSA (D); and corresponding time-resolved current profiles from all 100 pixels after 10 steps (E), enabling spatiotemporal reconstruction of object motion. (**F** to **H**) In-sensor motion perception of vectorized patterns under complex dynamic conditions. Initial blank frame without input (F); photocurrent mapping during left-to-right motion under 532 nm illumination (G); and mapping of leaving motion pattern under the same light stimulus (H).

Due to the device’s pronounced nonlinear conductance accumulation under optical stimulations, it is suitable for optical encoding applications. When tested under high-frequency optical pulses (100 Hz, 532 nm, 1.27 mW·cm^−2^), as shown in Fig. 4A, the Au@MoS_2_ device exhibits a notable photoresponse with increasing conductance that eventually saturates. Furthermore, its 5-bit binary optical encoding capability was evaluated (Fig. 4B). In cases where “1” pulses are continuous without “0” intervals, the responses are nearly identical. However, when “0” intervals are inserted between “1” pulses, the device shows a substantially reduced conductance, producing a unique stepwise response. This behavior originates from the dynamic trapping/release of carriers modulated by pulse timing, which is crucial for implementing spatiotemporal encoding in neuromorphic systems.

To clarify how this pulse-timing-dependent behavior differs from conventional long-exposure integration, we schematically compare the effective temporal weighting in the two cases (fig. S8). In conventional long-exposure imaging, consecutive optical inputs are approximately integrated with uniform temporal weight within the exposure window. In contrast, the Au@MoS_2_ PNSA exhibits a non-uniform temporal weighting governed by relaxation dynamics, such that earlier inputs decay more strongly while later inputs contribute more prominently to the final readout. As a result, unlike conventional long-exposure accumulation that mainly reflects motion occupancy, the PNSA-based fusion can preserve recency-dependent intensity gradients and retain temporal ordering information in a single fused output. This relaxation-dependent temporal fusion mechanism provides the physical basis for dynamic motion perception and trajectory-sensitive visual encoding.

To explore the wavelength universality of optical programming, fig. S9 presents the responses of the Au@MoS_2_ devices under different light pulse stimuli at 625 nm, 532 nm, and 455 nm. For each wavelength, the device realizes stepwise current responses corresponding to combinations of optical pulses (encoded as 4-bit binary sequences). The current escalates with the number of “1” bits in the coding sequence, demonstrating that the Au@MoS_2_ device maintains robust optical encoding performance across a broad spectral range. Notably, this behavior contrasts sharply with the response of non-core-shell MoS_2_ optoelectronic synaptic devices (fig. S10). In non-core-shell devices, the conductance response lacks the clear stepwise modulation observed in Au@MoS_2_ devices, which is attributed to the weakened plasmon-enhanced light-matter interaction and limited carrier trapping efficiency. These results highlight the superior optical encoding performance of the Au@MoS_2_ device, stemming from the synergistic plasmonic nanofocusing and defect-mediated carrier dynamics in the core-shell heterostructure, and its potential in advanced image processing and dynamic vision applications.

**Fig. 5.**
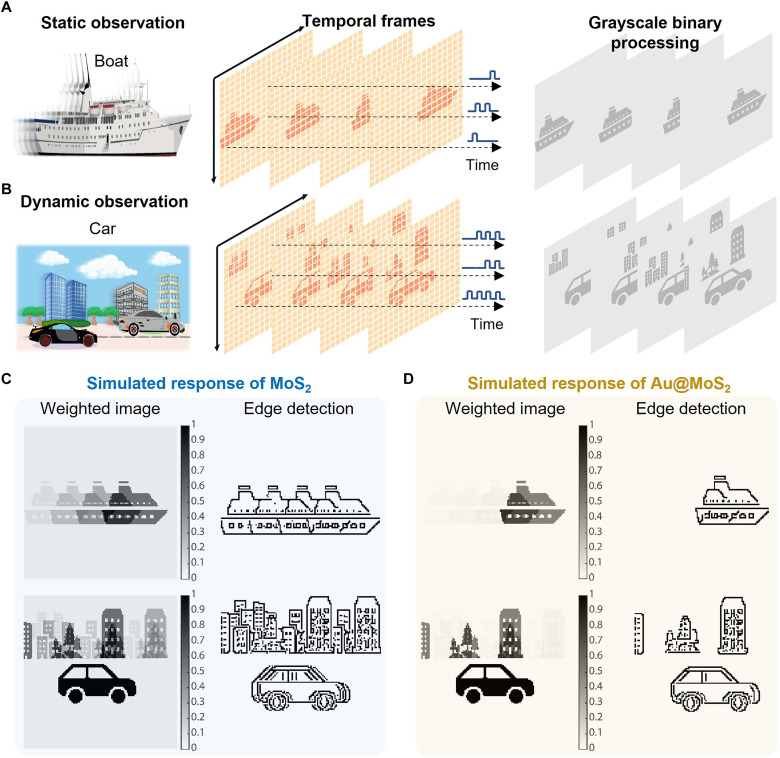
PNSA-based dynamic tracking simulation. (**A** and **B**) Temporal-frame sampling of fast-moving ship (A) and car (B) targets on a 128 × 128 synaptic arrays. (**C** and **D**) Weighted-image and edge-detection outputs reconstructed using the optical encoding behaviors of the non-core-shell MoS_2_ (C) and the Au@MoS_2_ PNSA (D).

Based on the enhanced optoelectronic response capability and rapid relaxation characteristics of Au@MoS_2_ devices, motion perception can be achieved by using the Au@MoS_2_ PNSA, as shown in Fig. 4C. Specifically, a trajectory composed of a moving pixel spot from step 1 to step 10 at constant velocity is predefined (Fig. 4D). After the motion ends, the current response of a 100-pixel-scale Au@MoS_2_ PNSA is recorded in a single shot, yielding complete motion trajectory information (Fig. 4E). Figure 4, F to H, further demonstrate in-sensor motion perception of vectorized patterns using a 32 × 32 Au@MoS_2_ PNSA under dynamically varying conditions. Building on the experimentally measured coded optical-pulse responses of the devices, we further performed simulations for fast-moving cars and ships in complex backgrounds. As illustrated in Fig. 5, A and B, temporal frame sequences of moving targets were mapped onto a 128 × 128 PNSA. In the simulation, the grayscale intensity of each pixel in the weighted image was assigned according to the measured current levels under 4-bit binary-coded optical pulse combinations. Figure 5, C and D, compare the simulated imaging results of non-core-shell MoS_2_ devices and the Au@ MoS_2_ PNSA. Compared with the non-core-shell MoS_2_ case, the Au@ MoS_2_ PNSA produces clearer weighted images and more accurate edge profiles under high-speed dynamic conditions. This improvement originates from the stronger coded-response contrast and more effective temporally weighted sensing enabled by the plasmon-enhanced light–matter interaction and efficient carrier dynamics in the Au@MoS_2_ core-shell heterojunction. These results highlight the potential of the Au@MoS_2_ PNSA for motion-sensitive imaging in dynamic environments.

**Fig. 6.**
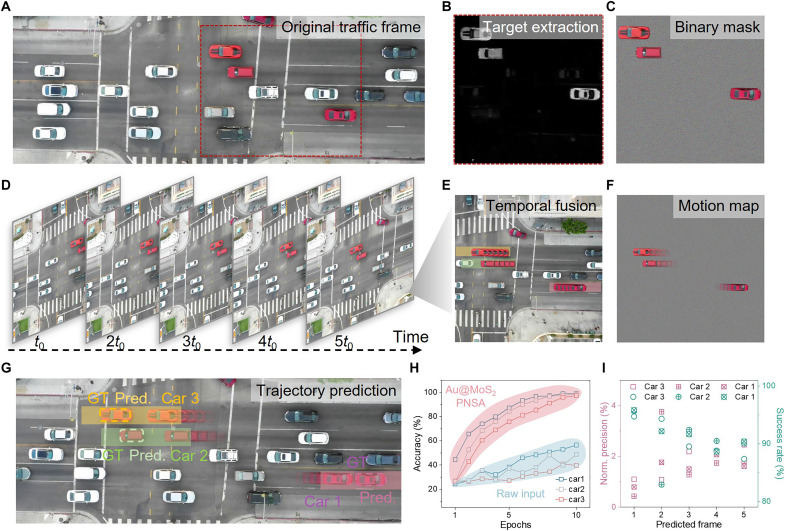
Intelligent motion perception and prediction based on the Au@MoS_2_ PNSA. (**A**) Raw traffic frame with selected traffic region. (**B**) Color-saturation-based extraction of the vehicles from the background. (**C**) Pixel-level binarization to generate the binary target mask used for subsequent temporal encoding. (**D**) Five consecutive traffic frames with a frame interval of step *t*_0_ = 0.2 s. (**E**) Relaxation-dependent temporal fusion of consecutive frames to generate an enhanced composite motion image. (**F**) Thresholded motion map highlighting vehicle contours and trajectories. (**G**) Predicted (Pred.) and ground-truth (GT) trajectories of three vehicles (Car 1–3) at future time steps. (**H**) Four-class motion-direction classification accuracy using a CNN for temporally encoded motion representation (Au@MoS_2_ PNSA) and raw frame inputs (Raw input). (**I**) Frame-wise trajectory-prediction evaluation for each vehicle, including normalized displacement error (Norm. Prec., left axis) and success rate (right axis).

To further demonstrate practical utility, we used a simulated Au@MoS_2_ PNSA for preprocessing traffic-surveillance video (Fig. 6A). Starting from the original traffic frame, color-saturation-based target extraction (Fig. 6B) and subsequent binarization (Fig. 6C) were applied to generate pixel-level temporal optical pulse sequences that encode the presence or absence of vehicle information across consecutive frames. These sequences were converted into a temporally encoded motion representation using the PNSA temporal weighting scheme (figs. S7 and S8), producing enhanced fused motion images and thresholded motion maps that clearly highlight vehicle contours and trajectories (Fig. 6, D to F). Based on the temporally encoded motion maps, the future trajectories of three vehicles were predicted using the positions, velocities, and accelerations estimated from the first five frames under a constant-acceleration motion model, and the predicted (Pred.) and ground-truth (GT.) trajectories are overlaid in Fig. 6G. The temporally encoded motion representation was subsequently used as the input to a CNN for four-class motion-direction classification. When evaluated using the temporally encoded motion maps as test inputs, the trained CNN achieved a motion-direction recognition accuracy of 98.95% after 10 epochs, whereas the corresponding baseline model using raw input exhibited substantially lower accuracy (Fig. 6H), underscoring the benefit of temporal encoding for downstream classification. The subsequent five-frame prediction yielded a normalized displacement error below 2% and a success rate exceeding 90% (Fig. 6I). These results demonstrate the potential of the Au@MoS_2_ PNSA-based temporal encoding framework for robust motion perception and short-horizon trajectory forecasting.

## DISCUSSION

In this work, we present a bioinspired plasmonic nanofocusing synaptic array (PNSA) that integrates oil-droplet-inspired light concentration with rapid optoelectronic signal processing for motion-sensitive visual sensing. By engineering a wafer-scale Au@MoS_2_ core-shell heterojunction with >99.5% spatial uniformity, we establish a materials platform that not only delivers ultrafast photoelectric response (2.2 ms) and 5-bit conductance programmability, but also scales to high-density arrays with exceptional device yield (100%) and uniformity (2.2% variation). The 32 × 32 PNSA enables generation of temporally encoded motion representations that support 98.95% accuracy in motion recognition after only 10 training epochs, demonstrating the promise of this bioinspired hardware framework for motion-sensitive visual perception. These features are further underscored by a quantitative comparison of our PNSA with the conventional approach and other representative neuromorphic vision sensors, as summarized in table S1. Our findings bridge micro-optical bioinspiration with large-scale optoelectronic integration, offering a powerful path toward high-speed, adaptive, and energy-efficient neuromorphic vision systems. This platform paves the way for intelligent sensing and on-chip decision-making in scenarios ranging from autonomous vehicles and aerial robotics to next-generation edge AI devices, where real-time recognition under dynamic conditions is imperative.

## MATERIALS AND METHODS

### Preparation of Au nanoparticles

A 2-inch tube furnace is used for annealing, with the wafer sample placed at the center of the heating zone of the tube furnace. The heating program is set as follows: a heating rate of 20°C/min, an annealing temperature of 550°C, and a holding time of 6 minutes. Under high-temperature conditions, the Au film melts and forms uniformly distributed Au nanoparticles.

### Synthesis of Au@MoS_2_ core-shell heterojunctions

The growth of Au@MoS_2_ core-shell heterojunction films is accomplished using a three-zone CVD system. During the growth process, sulfur powder (S, Alfa Aesar, 99.5%) and molybdenum trioxide (MoO_3_, Alfa Aesar, 99.999%) serve as reaction precursors respectively, while the patterned and annealed silicon wafers (SiO_2_/Si) act as the growth substrate. A precursor supply strategy with spatial separation of Mo and S sources is employed: sulfur powder and MoO_3_ are placed in separate quartz tubes, with independent temperature zone control. A sufficient amount of sulfur powder is placed in a quartz tube upstream of the CVD system, located in the first temperature zone (*T*_1_ = 180°C), with 100 sccm Ar as the carrier gas for the sulfur source. For Mo source supply, a molecular sieve/MoO_3_ composite structure in a segmented quartz tube is used, situated in the second temperature zone (*T*_2_ = 580°C), and the carrier gas for the molybdenum source consists of 48 sccm Ar and 2 sccm O_2_. The substrate is placed in the third temperature zone (*T*_3_ = 645°C). The holding time after the start of the reaction is set to 15 min, and the reaction pressure is 300 mTorr.

### Fabrication and integration of devices

The Au@MoS_2_ core-shell heterojunction samples are subjected to photolithography and Reactive Ion Etching (RIE) technology to fabricate a 32 × 32 Au@MoS_2_ core-shell heterojunction array. First, the prepared sample undergoes the first Laser Direct Writing (LDW) treatment, where the heterojunction region is protected by a photoresist film, while excess material is exposed. The sample is then processed using RIE to remove the material surrounding the heterojunction. We use a combination of SF_6_ and Ar as etching gases. A relatively low RF power of 20 W is applied to reduce potential damage to materials. The gas flow rates are maintained at 20 sccm for both SF_6_ and Ar and the etching time is precisely controlled at 40 s. Next, an alignment lithography step is performed, followed by thermal evaporation to deposit 10 nm of Cr and 50 nm of Au as the first layer of electrodes onto the exposed patterns. Afterward, another alignment lithography step is carried out at the source-drain electrode intersections, and 100 nm of Si_3_N_4_ is deposited as an insulating layer. A final alignment lithography step is then performed, and 20 nm of Cr and 180 nm of Au are deposited as the second layer of electrodes onto the exposed patterns. Through this process, the Au@MoS_2_ core-shell heterojunction array devices are successfully fabricated. Finally, the array devices are integrated onto a Printed Circuit Board (PCB) by wire bonding.

### Characterizations and measurements

An optical microscope (OM) (BX51, OLYMPUS) is used to characterize the morphology of Au@MoS_2_ heterojunctions. A field-emission scanning electron microscope (SEM) (Quanta650 FEG, FEI) measures the morphology and composition. The thicknesses of Au@MoS_2_ heterojunctions are acquired by an atomic force microscope (AFM) (Dimension Icon, Bruker). The Raman spectra and Raman mapping of heterojunctions are obtained on a confocal Raman spectroscopy (WITec Alpha 300 Raman) with a 532 nm excitation laser at 2 mW. The PL spectra of and PL mapping of heterojunctions are obtained with a 532 nm excitation laser at 5 mW. The absorption spectra are recorded by a *UV-Vis* spectrophotometer (jasco msv5200). The x-ray diffraction (XRD) (Rigaku Rapid IIR, Rigaku) and x-ray photoelectron spectroscopy (XPS) (AXIS SUPRA+, Shimadzu) are used to obtain the composition and chemical states. The crystal structure of heterojunctions were further characterized by transmission electron microscopy (TEM) (Thermo-Fisher, Talos F200S). The photoelectric measurements are based on the probe station (Lake Shore, TTPX) equipped with a semiconductor device analyzer (Agilent, B1500A). Wavelength of the laser source is 532 nm, where the incident power density is adjustable. All the photoelectric measurements were performed at room temperature and under ambient pressure. The image sensor chip testing is conducted using a custom-built system consisting of a multiplexer, a voltage source, a laptop, and LabVIEW software. The voltage source (PXle-4135, National Instruments) and multiplexer (NI TB-2630B, National Instruments) are housed within a chassis (PXle-1088, National Instruments).

### PNSA-based dynamic tracking and motion prediction simulation

For the dynamic-tracking process in Fig. 5, fast-moving ship and car targets were represented as temporal frame sequences on a 128 × 128 synaptic array. Pixel grayscale values in the weighted images were assigned according to the experimentally measured current responses of the devices to 4-bit binary-coded optical pulse combinations. For the Au@MoS_2_ PNSA case, the lookup-table weights were derived from the 4-bit coded-pulse responses at 532 nm in fig. S9, whereas the non-core-shell MoS_2_ case used the corresponding responses in fig. S10. These current levels were mapped onto the target frames to reconstruct temporally weighted images, from which edge-detection outputs were extracted to compare dynamic-imaging fidelity under high-speed motion.

For the traffic-video analysis in Fig. 6, Python was used to preprocess consecutive frames by color-based target extraction and binarization, and to convert pixel-level target information into temporal optical pulse sequences. These sequences were then transformed into temporally encoded motion representations based on the device characteristics in figs. S7 and S8, following the experimentally observed pulse-order dependence and relaxation behavior of the Au@MoS_2_ device. The resulting motion maps were further thresholded to highlight vehicle contours and trajectories. For motion-direction recognition, the encoded motion maps were used as inputs to a CNN for four-class classification (forward, backward, left, and right). For trajectory prediction, the positions, velocities, and accelerations of the target vehicles were estimated from the first five frames, and a constant-acceleration model was used to predict the subsequent five frames. Prediction performance was evaluated by the normalized displacement error, defined as the Euclidean distance between predicted and ground-truth centers normalized by the diagonal length of the target box, and by the success rate, calculated from the intersection-over-union (IoU) between the predicted and ground-truth boxes.

## Supplementary Materials

### This PDF file includes:

Fig. S1 to S10 Table S1 References
