# Supplementary material for: Scalable nanofocusing heterojunctions for raptor-inspired on-chip vision perception
Source: Sci Adv. 2026 Jul 23;12(30):eaee7840. doi: 10.1126/sciadv.aee7840 (PMC13394458; doi:10.1126/sciadv.aee7840)
Supplement: Supplementary file 1 [file sciadv.aee7840_sm.pdf]

Science Advances
AAAS

Supplementary Materials for

Scalable nanofocusing heterojunctions for raptor-inspired on-chip vision perception

Lanhao Qin *et al.*

Corresponding author: Wenhua Hu, whu10@whut.edu.cn; Yuan Li, yuanli1@hust.edu.cn;
Yang Chai, ychai@polyu.edu.hk; Tianyou Zhai, zhaity@hust.edu.cn

Sci. Adv. **12**, eaee7840 (2026)
DOI: 10.1126/sciadv.aee7840

This PDF file includes:

Fig. S1 to S10
Table S1
References

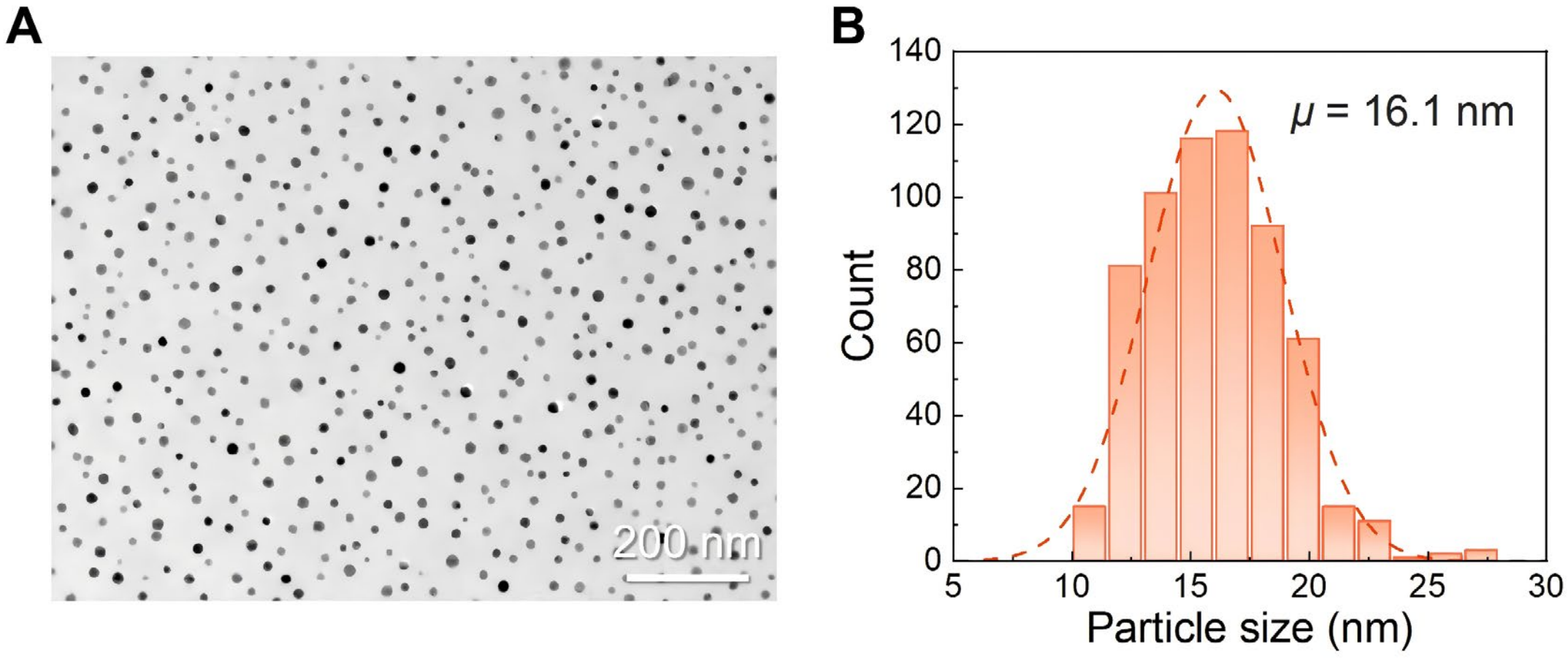

Fig. S1. Microscopic morphology characterization of Au@MoS_2 nanoparticles. (**A**) SEM image of Au@MoS_2 nanoparticles. (**B**) Statistical graph of particle size distribution of Au@MoS_2 nanoparticles in the region.

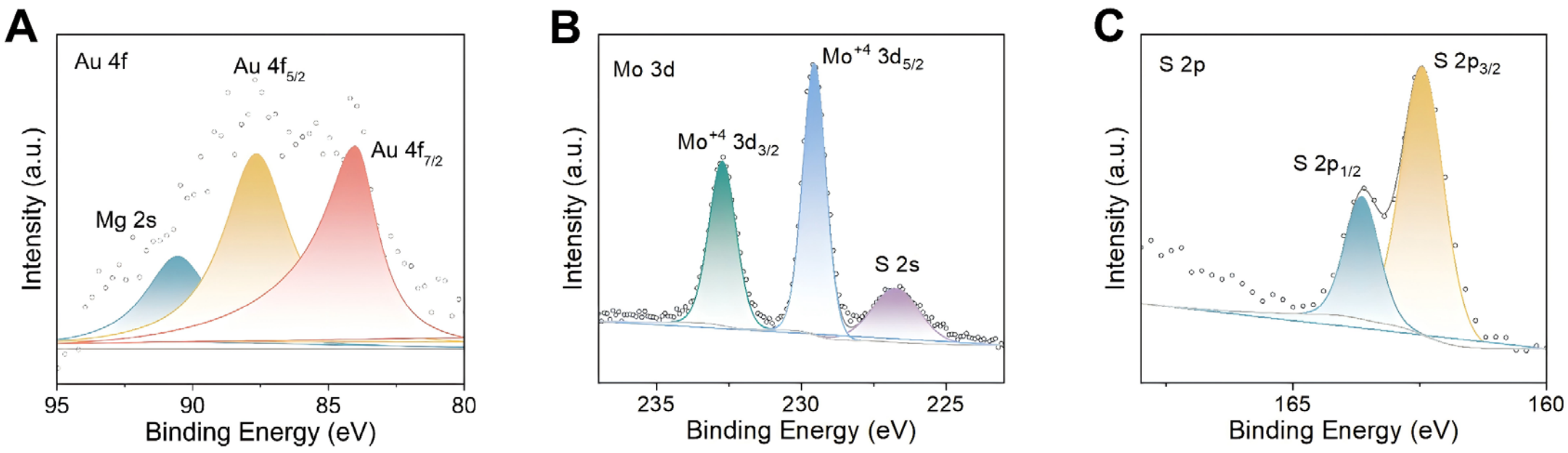

Fig. S2. XPS spectra of Au@MoS_2 heterojunctions. (**A**) XPS spectra of Au element. (**B**) XPS spectra of Mo element. (**C**) XPS spectra of S element.

High-resolution XPS is used to conduct a detailed analysis of the chemical composition of Au@MoS_2 samples. Fig. S2A shows the peaks of the Au 4f orbital at 87.70 eV (Au $4f_{5/2}$) and 84.05 eV (Au $4f_{7/2}$). Fig. S2B shows that in the Mo 3d spectrum, peaks appear at 229.81 eV and 232.85 eV, corresponding to the Mo $3d_{5/2}$ and Mo $3d_{3/2}$ orbitals, respectively. Fig. S2C shows two peaks in the S 2p spectrum at 162.60 eV (S $2p_{1/2}$) and 163.91 eV (S $2p_{3/2}$). These fitting results are consistent with previous research reports on MoS_2 and Au.

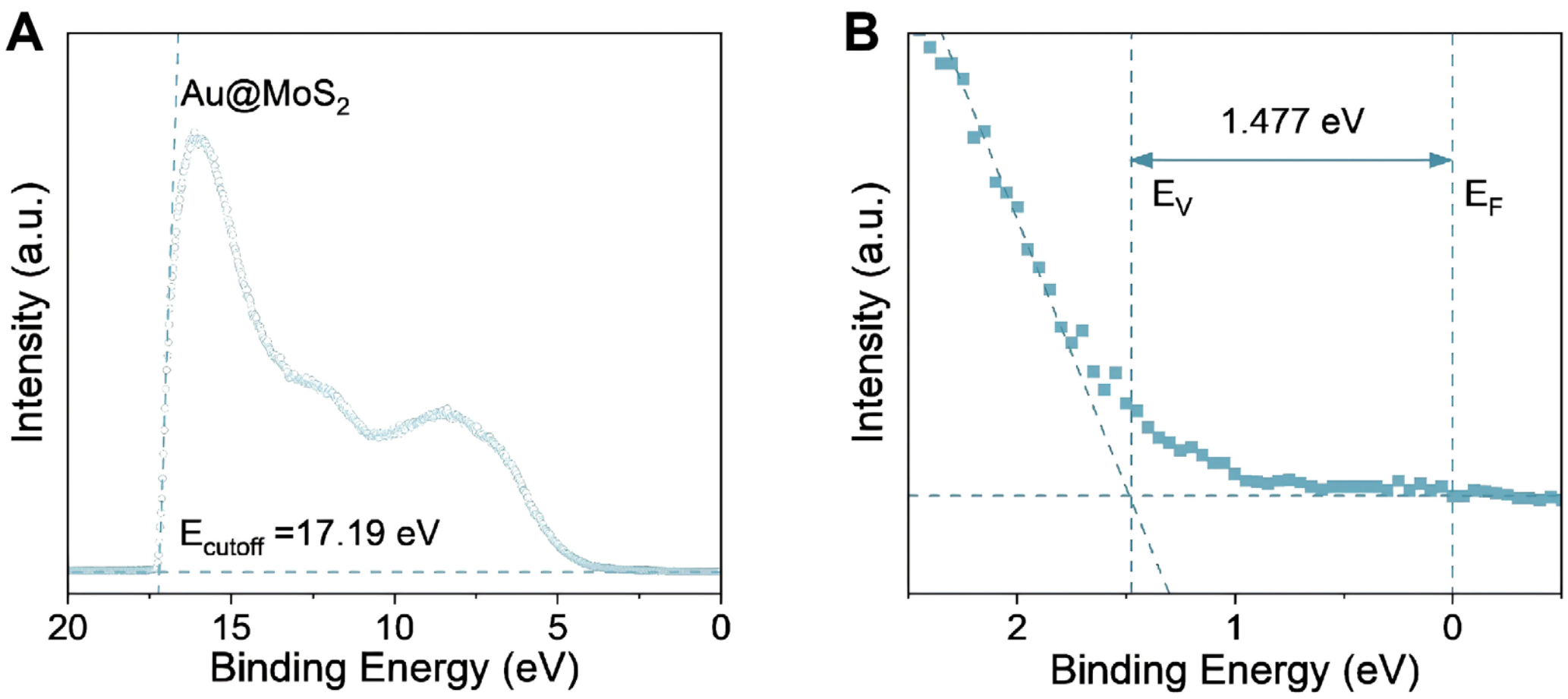

Fig. S3. UPS characterization of Au@MoS_2 heterojunctions. (**A**) UPS spectrum of the secondary electron cutoff region. (**B**) UPS spectrum of the valence band region.

The energy band structure of Au@MoS_2 heterojunctions is characterized by ultraviolet photoelectron spectroscopy (UPS). The work function (W) of the sample is calculated using the formula: $W = hv - E_{cutoff}$, where hv is the photon energy of the He I light source (21.2 eV), and E_{cutoff} is the secondary electron cutoff energy. From the fitting results in Fig. S3A, the cutoff energy is determined to be 17.19 eV. According to the formula, the work function of the Au@MoS_2 heterojunction is calculated as 4.01 eV, indicating that the Fermi level is located at −4.01 eV relative to the vacuum level (E_{vac} = 0 eV). Furthermore, by linearly extrapolating the low-binding-energy region to the baseline (Fig. S3B), the fitting results show that the difference between the valence band maximum (E_{VBM}) and the Fermi level is 1.477 eV. Based on this, the E_{VBM} of the Au@MoS_2 heterojunction film is further extrapolated to be −5.487 eV.

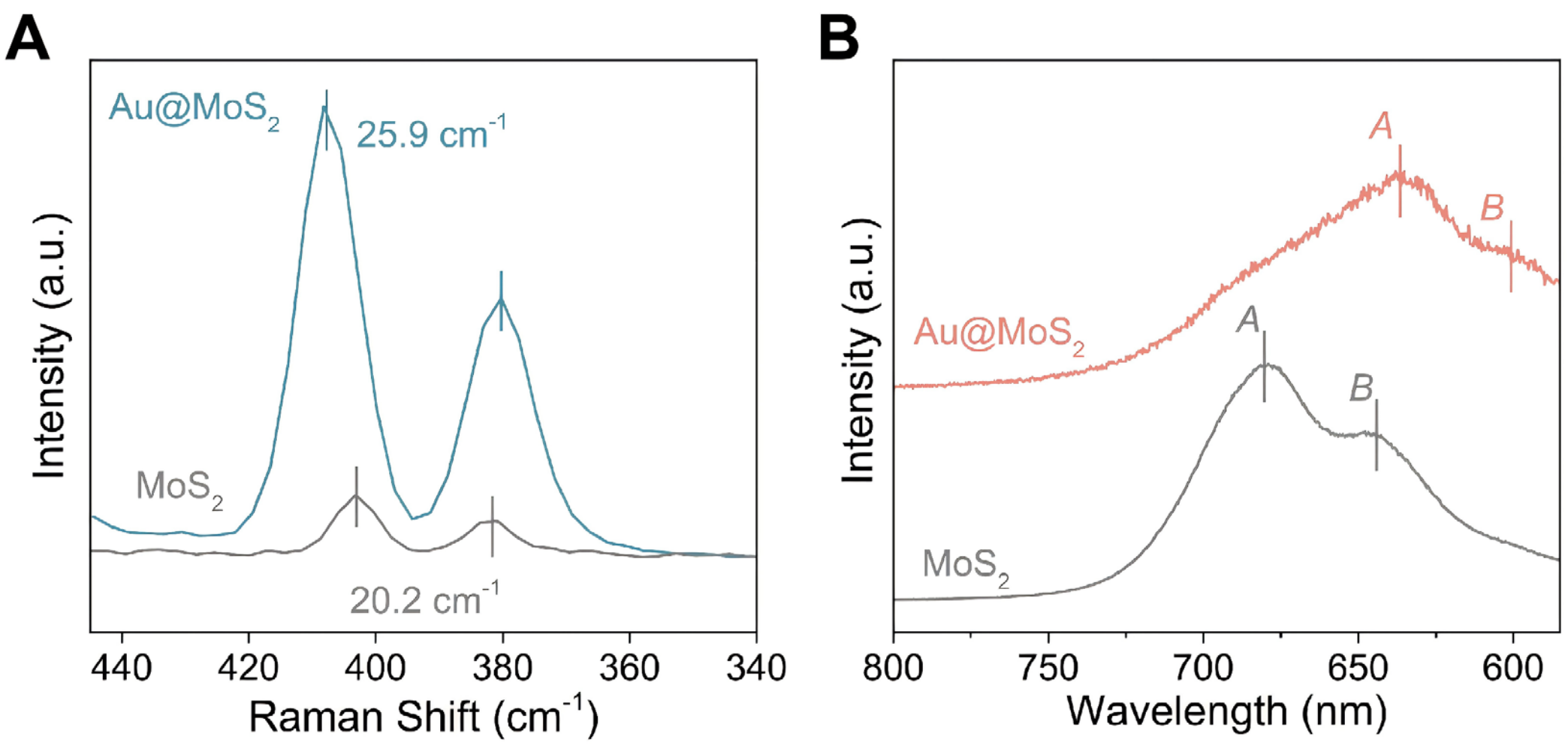

Fig. S4. Optical characterization of Au@MoS_2 heterojunctions. (**A**) Comparison of Raman spectra between Au@MoS_2 and MoS_2. (**B**) Comparison of PL spectra between Au@MoS_2 and MoS_2.

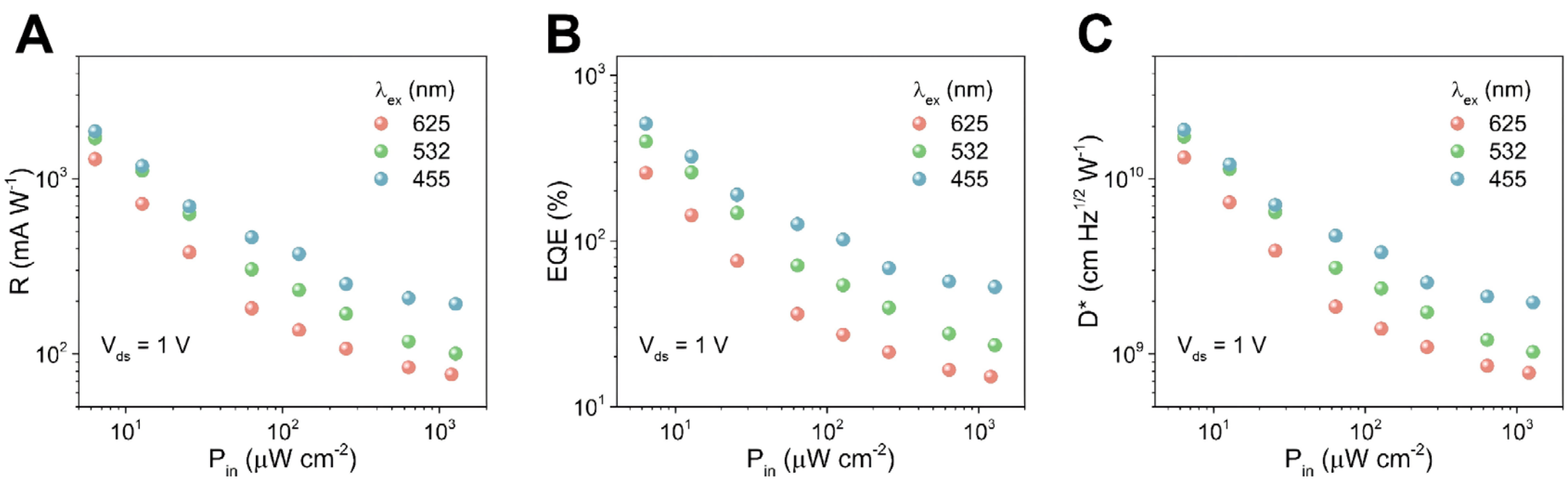

Fig. S5. Response characteristics of Au@MoS_2 brain-inspired visual sensors to incident light of different powers. (A) Responsivity (R); (B) External quantum efficiency (EQE); (C) Detectivity (D*).

To further evaluate the optoelectronic response characteristics of Au@MoS_2 brain-inspired visual sensors, this study tests the effects of different optical power densities (P_{in}) and different incident wavelengths (455 nm, 532 nm, and 625 nm) on device performance parameters under the condition of V_{ds} = 1 V, and calculates the device's responsivity (R), external quantum efficiency (EQE), and specific detectivity (D*) under different conditions. Fig. S5A shows the relationship between the device's R and P_{in}. It can be observed that R gradually decreases with increasing P_{in}, with the maximum responsivity of the device reaching 1.71×10^3 mA/W. This indicates that the Au@MoS_2 array devices possess extremely high photosensitivity under low optical power density conditions. Fig. S5B shows that EQE exhibits a gradual decreasing trend with increasing P_{in}, reaching a maximum value of 466.5% under the conditions of 455 nm and 6.37 μW/cm^2. Notably, a value exceeding 100% usually indicates the presence of a photoelectric gain effect, suggesting that there may be an efficient multiple carrier recycling mechanism in the device. Fig. S5C shows the relationship between the

device's D* and P_{in}. Test results indicate that the Au@MoS_2 array sensor has extremely high detectability under low optical power density, with the maximum specific detectivity reaching 1.74×10^{10} Jones. These results confirm that the brain-inspired visual sensor constructed in this study, which is based on large-scale patterned Au@MoS_2 film growth, possesses excellent photoresponse performance and a potential photoelectric gain mechanism, exhibiting great application potential in the fields of image perception and dynamic visual information processing.

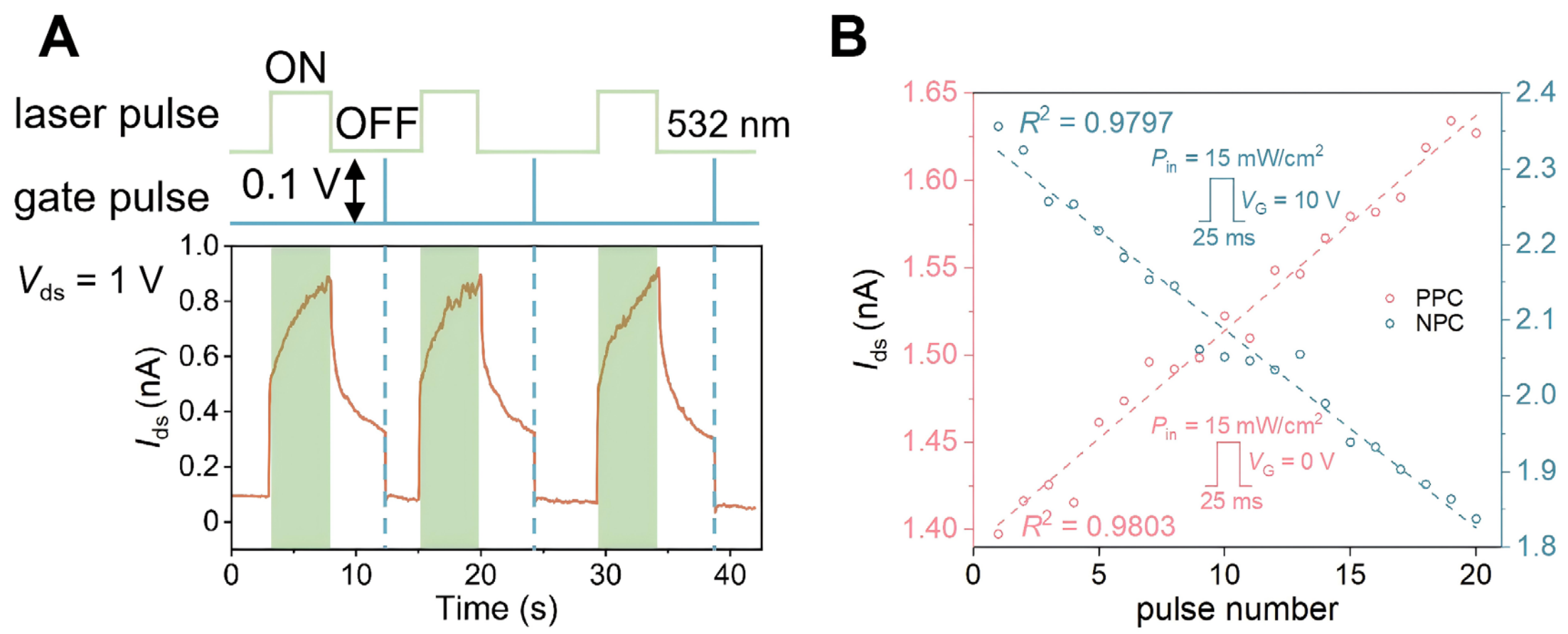

Fig. S6. Photoelectric coordinated control for Au@MoS_2 devices. (**A**) Current response to light/dark stimuli cycles using V_{ds} = 1 V, with the green area representing continuous laser pulses and the blue dashed line representing 25 ms gate voltage pulses. (**B**) PPC and NPC behaviors of response currents versus the number of gate voltage pulses.

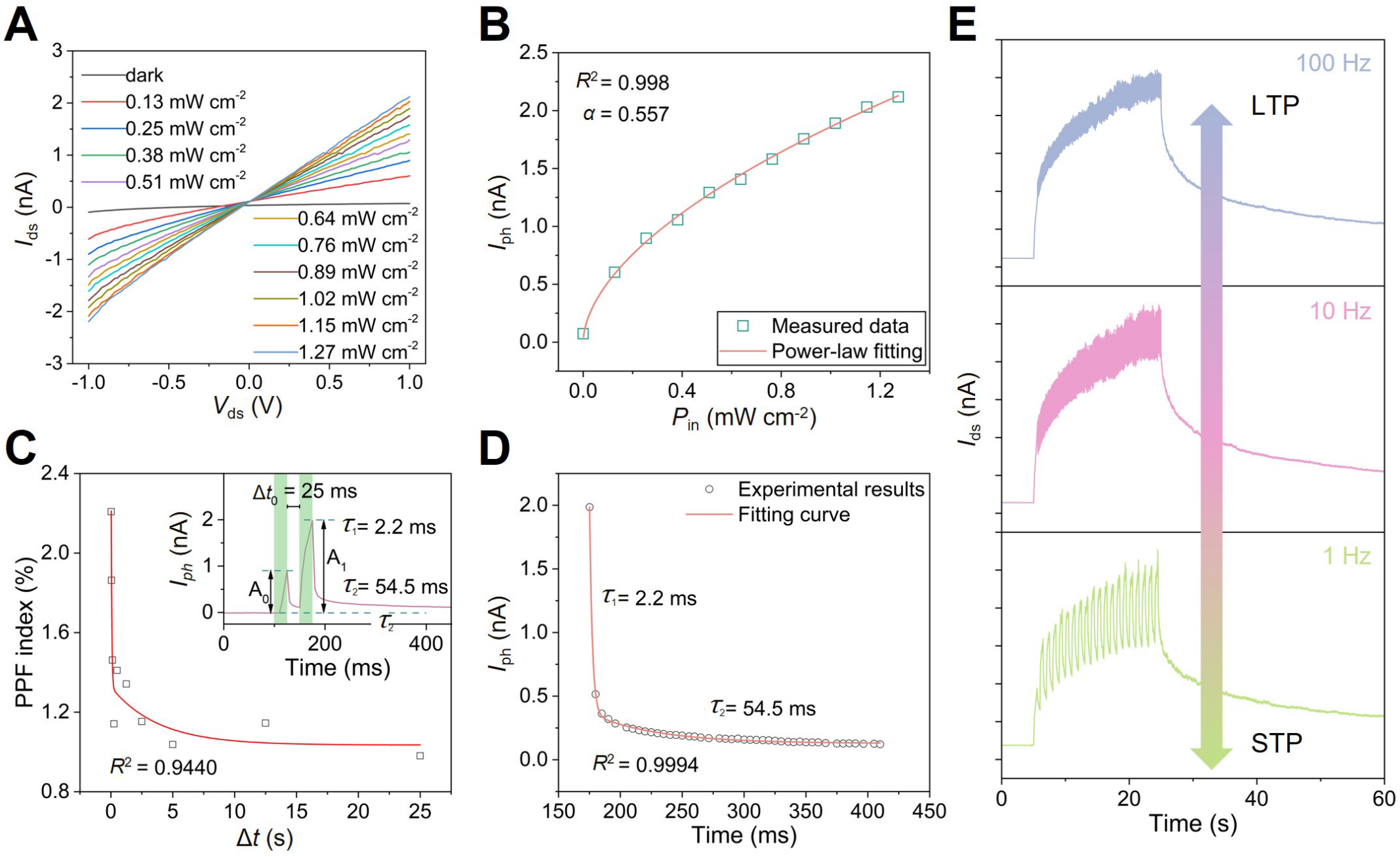

Fig. S7. Optoelectronic synaptic characteristics of the Au@MoS_2 device. (**A**) *I-V* curves under dark conditions and various light power densities. (**B**) Power-law fitting of the photocurrent as a function of light intensity. (**C**) Paired-pulse facilitation (PPF) index as a function of inter-pulse interval, along with the corresponding fitting curve. (**D**) Decay characteristics of the photocurrent under dual-pulse stimulation at 20 Hz. (**E**) Short-term to long-term plasticity (STP-LTP) transition behavior under different optical pulse frequencies.

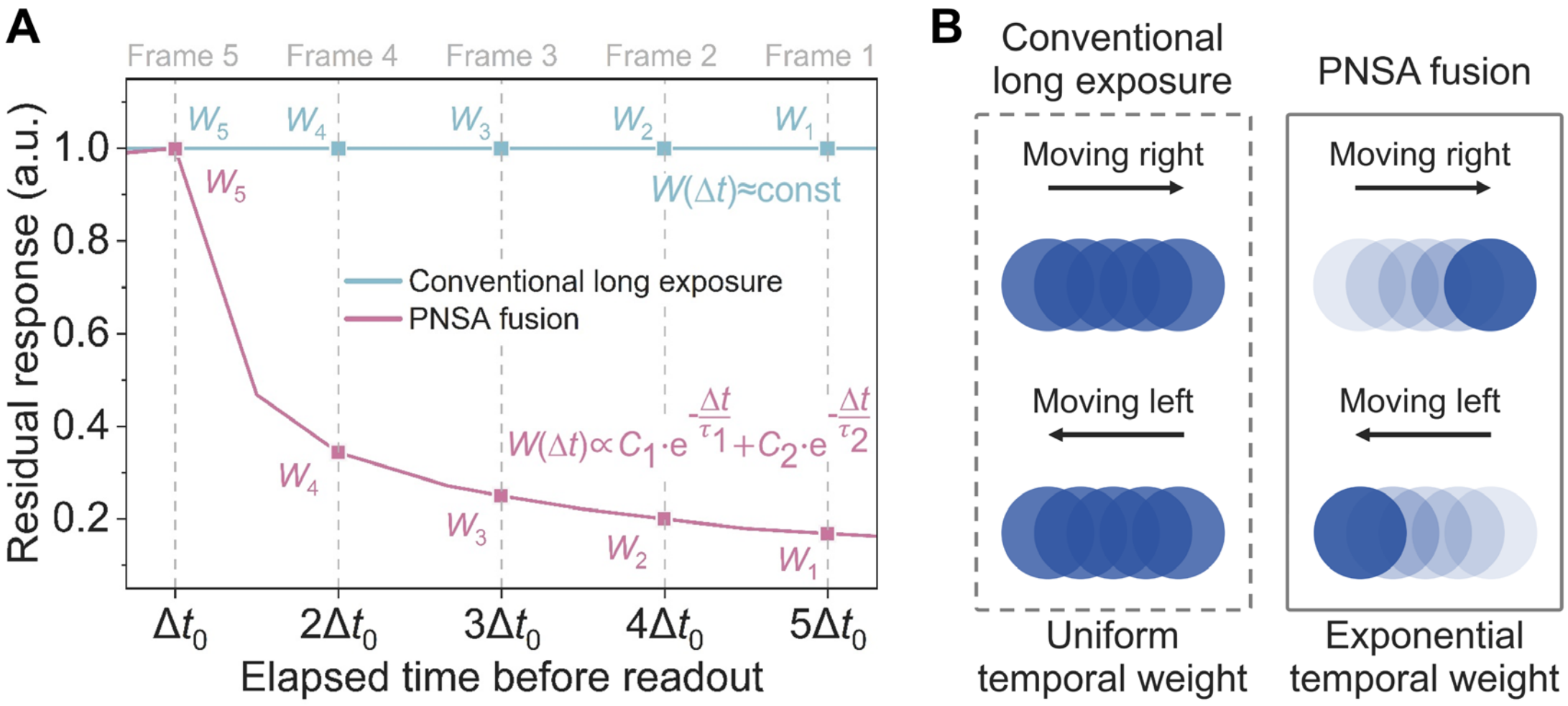

Fig. S8. Comparison between conventional long-exposure imaging and PNSA-based fusion. (**A**) Schematic comparison of the effective temporal weighting assigned to consecutive frames before readout. In conventional long-exposure imaging, signals within the exposure window are integrated with approximately uniform temporal weight. In contrast, the PNSA exhibits a non-uniform temporal weighting governed by device relaxation, in which earlier frames contribute less strongly to the final readout than later frames. The PNSA weighting is illustrated using a bi-exponential decay form consistent with the experimentally observed relaxation behavior. (**B**) Schematic illustration of fused motion traces for opposite motion directions along the same path. Under conventional long exposure, consecutive positions are accumulated with nearly uniform intensity, mainly reflecting motion occupancy. By contrast, PNSA-based fusion produces a recency-dependent intensity gradient, such that the latest positions appear stronger than earlier ones. As a result, the fused output preserves temporal ordering and distinguishes motion direction in a single readout.

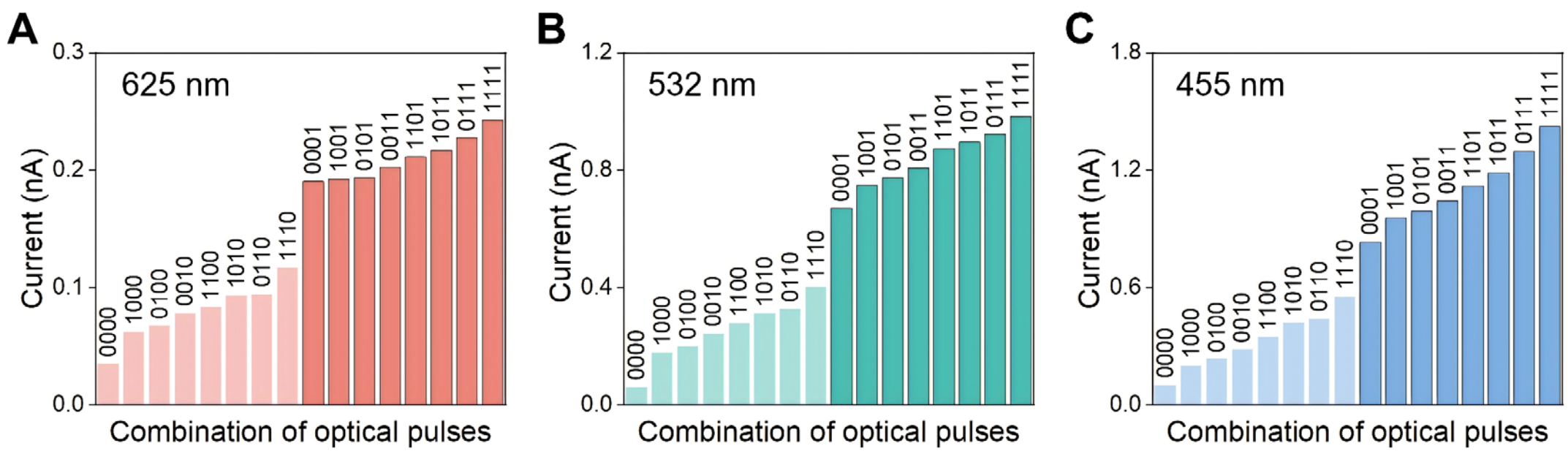

Fig. S9. Current response of the Au@MoS_2 optoelectronic synaptic device to 4-bit binary-coded optical pulses. (A) Response under 625 nm light. (B) Response under 532 nm light. (C) Response under 455 nm light.

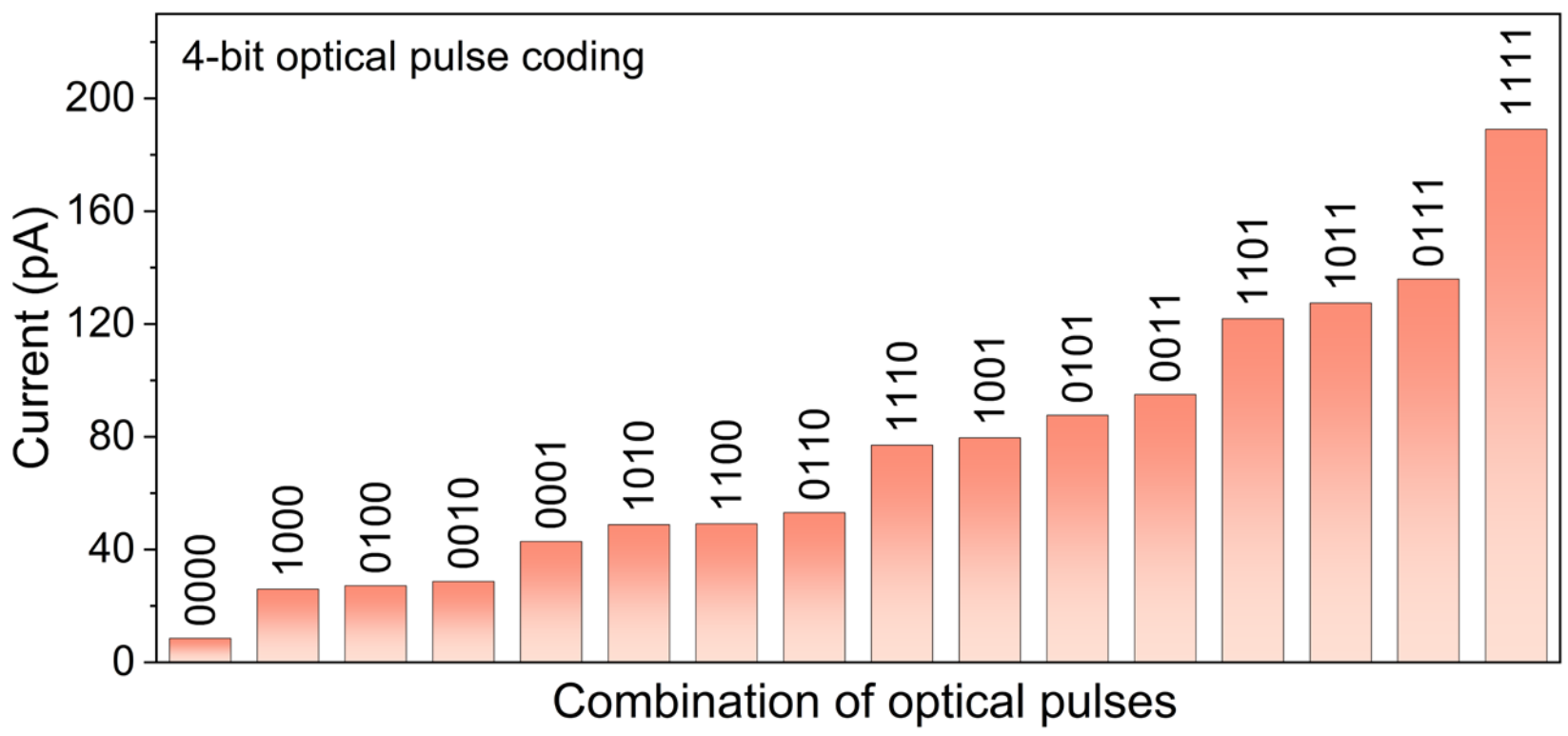

Fig. S10. Current response of the non-core-shell structured MoS_2 optoelectronic synaptic device to 4-bit binary-coded optical pulses.

Table S1. Performance comparison between Au@MoS_2 PNSA, the conventional approach, and other neuromorphic vision sensors.

Performance Metrics	This Work (Au@MoS_2 PNSAs)	Other Neuromorphic Vision Sensors	Conventional Approach
Device-to-device variation	2.2%	5%~10%	Commercial microlens array (3%~5%) + CMOS (2%~3%)
Relaxation time	2.2 ms	5~10 ms	>10 ms
Theoretical refresh rate	~ 450 Hz	100~200 Hz	<200 Hz
Motion direction recognition accuracy	98.95% (10 training epochs)	92%~95% (50~80 training epochs)	~90% (>100 training epochs)
Operating current range	pA~nA	nA~μA	μA~mA
System architecture	Monolithic on-chip integration	Partial integration + Independent DSP	Microlens Array + Si CMOS + Independent DSP
Energy consumption	On-chip processing (Ultra-low power)	Sensing + off-chip transmission (Low power)	Off-chip data transfer + DSP heavy lifting (High power)
Application scenarios	High-speed dynamic vision scenarios	General imaging, low-speed static/quasi-dynamic visual recognition	Conventional static/low-speed visual scenarios

Note: Representative values collected from Refs. (*11, 24, 36–48*).

REFERENCES

1. B. Ouyang, J. Wang, G. Zeng, J. Yan, Y. Zhou, X. Jiang, B. Shao, Y. Chai, Bioinspired in-sensor spectral adaptation for perceiving spectrally distinctive features. *Nat. Electron.* **7**, 705–713 (2024).

2. Y. Chen, M. Nazhamaiti, H. Xu, Y. Meng, T. Zhou, G. Li, J. Fan, Q. Wei, J. Wu, F. Qiao, L. Fang, Q. Dai, All-analog photoelectronic chip for high-speed vision tasks. *Nature* **623**, 48–57 (2023).

3. S. Feng, H. Sun, X. Yan, H. Zhu, Z. Zou, S. Shen, H. X. Liu, Dense reinforcement learning for safety validation of autonomous vehicles. *Nature* **615**, 620–627 (2023).

4. L. Qin, P. Guan, J. Shao, Y. Xiao, Y. Yu, J. Su, C. Zhang, Y. Li, S. Liu, P. Li, D. Ouyang, W. He, F. Liu, K. Zhu, K. Liu, Z. Yao, J. Wu, Y. Zhao, H. Li, F. Hui, P. Lin, M. Lanza, Y. Li, T. Zhai, Molecular crystal memristors. *Nat. Nanotechnol.* **20**, 1641–1647 (2025).

5. S. Goossens, G. Navickaite, C. Monasterio, S. Gupta, J. J. Piqueras, R. Pérez, G. Burwell, I. Nikitskiy, T. Lasanta, T. Galán, E. Puma, A. Centeno, A. Pesquera, A. Zurutuza, G. Konstantatos, F. Koppens, Broadband image sensor array based on graphene–CMOS integration. *Nat. Photonics* **11**, 366–371 (2017).

6. R. Chen, H. Shu, B. Shen, L. Chang, W. Xie, W. Liao, Z. Tao, J. E. Bowers, X. Wang, Breaking the temporal and frequency congestion of LiDAR by parallel chaos. *Nat. Photonics* **17**, 306–314 (2023).

7. B. Li, Q. Lin, M. Li, Frequency–angular resolving LiDAR using chip-scale acousto-optic beam steering. *Nature* **620**, 316–322 (2023).

8. Z. Sun, J. Liu, N. Zhang, W. Shen, C. Hu, L. Li, F. Yan, F. Xia, H. Li, Y. Li, T. Zhai, Enhanced in-plane polarization in two-dimensional $GaInS_3$ via strain engineering for self-powered photodetector. *Trans. Mater. Res.* **1**, 100009 (2025).

9. H. Jang, C. Liu, H. Hinton, M.-H. Lee, H. Kim, M. Seol, H.-J. Shin, S. Park, D. Ham, An atomically thin optoelectronic machine vision processor. *Adv. Mater.* **32**, e2002431 (2020).

10. L. Bian, Z. Wang, Y. Zhang, L. Li, Y. Zhang, C. Yang, W. Fang, J. Zhao, C. Zhu, Q. Meng, X. Peng, J. Zhang, A broadband hyperspectral image sensor with high spatio-temporal resolution. *Nature* **635**, 73–81 (2024).

11. Z. Wang, T. Wan, S. Ma, Y. Chai, Multidimensional vision sensors for information processing. *Nat. Nanotechnol.* **19**, 919–930 (2024).

12. A. Dodda, D. Jayachandran, A. Pannone, N. Trainor, S. P. Stepanoff, M. A. Steves, S. S. Radhakrishnan, S. Bachu, C. W. Ordonez, J. R. Shallenberger, J. M. Redwing, K. L. Knappenberger, D. E. Wolfe, S. Das, Active pixel sensor matrix based on monolayer MoS_2 phototransistor array. *Nat. Mater.* **21**, 1379–1387 (2022).

13. M. Lee, G. J. Lee, H. J. Jang, E. Joh, H. Cho, M. S. Kim, H. M. Kim, K. M. Kang, J. H. Lee, M. Kim, H. Jang, J.-E. Yeo, F. Durand, N. Lu, D.-H. Kim, Y. M. Song, An amphibious artificial vision system with a panoramic visual field. *Nat. Electron.* **5**, 452–459 (2022).

14. F. Liao, Z. Zhou, B. J. Kim, J. Chen, J. Wang, T. Wan, Y. Zhou, A. T. Hoang, C. Wang, J. Kang, J.-H. Ahn, Y. Chai, Bioinspired in-sensor visual adaptation for accurate perception. *Nat. Electron.* **5**, 84–91 (2022).

15. J. Chen, Z. Zhou, B. J. Kim, Y. Zhou, Z. Wang, T. Wan, J. Yan, J. Kang, J.-H. Ahn, Y. Chai, Optoelectronic graded neurons for bioinspired in-sensor motion perception. *Nat. Nanotechnol.* **18**, 882–888 (2023).

16. H. L. Payne, D. Aronov, Remote activation of place codes by gaze in a highly visual animal. *Nature* **643**, 1037–1043 (2025).

17. G. R. Martin, Avian vision. *Curr. Biol.* **32**, R1079–R1085 (2022).

18. S. Potier, M. Mitkus, A. Kelber, Visual adaptations of diurnal and nocturnal raptors. *Semin. Cell Dev. Biol.* **106**, 116–126 (2020).

19. D. G. Stavenga, B. D. Wilts, Oil droplets of bird eyes: Microlenses acting as spectral filters. *Philos. Trans. R. Soc. Lond. B Biol. Sci.* **369**, 20130041 (2014).

20. S. R. Young, G. R. Martin, Optics of retinal oil droplets: A model of light collection and polarization detection in the avian retina. *Vision Res.* **24**, 129–137 (1984).

21. D. Williams, Eagle eyed or bird brained? *Eye* **37**, 2426–2430 (2023).

22. H. M. Schryver, S. P. Mysore, Distinct neural mechanisms construct classical versus extraclassical inhibitory surrounds in an inhibitory nucleus in the midbrain attention network. *Nat. Commun.* **14**, 3400 (2023).

23. A. Asadollahi, E. I. Knudsen, Spatially precise visual gain control mediated by a cholinergic circuit in the midbrain attention network. *Nat. Commun.* **7**, 13472 (2016).

24. P. Xie, Y. Xu, J. Wang, D. Li, Y. Zhang, Z. Zeng, B. Gao, Q. Quan, B. Li, Y. Meng, W. Wang, Y. Li, Y. Yan, Y. Shen, J. Sun, J. C. Ho, Birdlike broadband neuromorphic visual sensor arrays for fusion imaging. *Nat. Commun.* **15**, 8298 (2024).

25. J. Zhou, F. Tian, J. Hu, Z. Shi, V. G. Godinez, D. P. Tsai, Z. Liu, Eagle-eye inspired meta-device for phase imaging. *Adv. Mater.* **36**, e2402751 (2024).

26. C. H. Brighton, L. N. Kloepper, C. D. Harding, L. Larkman, K. McGowan, L. Zusi, G. K. Taylor, Raptors avoid the confusion effect by targeting fixed points in dense aerial prey aggregations. *Nat. Commun.* **13**, 4778 (2022).

27. C. H. Brighton, G. K. Taylor, Hawks steer attacks using a guidance system tuned for close pursuit of erratically manoeuvring targets. *Nat. Commun.* **10**, 2462 (2019).

28. R. Liu, X. Zhu, S. Liu, D. Ouyang, X. Ma, F. Xia, Y. Yu, H. Zhang, J. Wu, S. Liu, W. Liang, Y. Li, T. Zhai, Interface-engineered Au@MoS_2 core-shell heterostructures with superior hot-carrier transfer dynamics for plasmonics and optoelectronics. *Sci. China Mater.* **66**, 3931–3940 (2023).

29. Y. Li, J. D. Cain, E. D. Hanson, A. A. Murthy, S. Hao, F. Shi, Q. Li, C. Wolverton, X. Chen, V. P. Dravid, Au@MoS_2 core–shell heterostructures with strong light–matter interactions. *Nano Lett.* **16**, 7696–7702 (2016).

30. J. M. Luther, P. K. Jain, T. Ewers, A. P. Alivisatos, Localized surface plasmon resonances arising from free carriers in doped quantum dots. *Nat. Mater.* **10**, 361–366 (2011).

31. Y. Deng, S. Liu, M. Li, N. Zhang, Y. Feng, J. Han, Y. Kapitonov, Y. Li, T. Zhai, Large-area growth of synaptic heterostructure arrays for integrated neuromorphic visual perception chips. *Chip* **3**, 100088 (2024).

32. Y. Deng, S. Liu, X. Ma, S. Guo, B. Zhai, Z. Zhang, M. Li, Y. Yu, W. Hu, H. Yang, Y. Kapitonov, J. Han, J. Wu, Y. Li, T. Zhai, Intrinsic defect-driven synergistic synaptic heterostructures for gate-free neuromorphic phototransistors. *Adv. Mater.* **36**, e2309940 (2024).

33. A. Park, R. Kantipudi, J. Göser, Y. Chen, D. Hao, N.-C. Yeh, Strongly enhanced electronic bandstructure renormalization by light in nanoscale strained regions of monolayer MoS_2/Au(111) heterostructures. *ACS Nano* **18**, 29618–29635 (2024).

34. L. Dong, J. Yang, X. Yue, S. Dong, W. Li, X. Li, Defect engineering of MoS_2 nanosheets by heavy-ion irradiation for hydrogen evolution. *ACS Appl. Nano Mater.* **6**, 18858–18868 (2023).

35. H. Liu, A. T. Neal, Z. Zhu, Z. Luo, X. Xu, D. Tománek, P. D. Ye, Phosphorene: An unexplored 2D semiconductor with a high hole mobility. *ACS Nano* **8**, 4033–4041 (2014).

36. Y. Kim, H. Park, M. Kim, S. Jang, D. Y. Jeong, L. S. Handriani, H. Yun, N. Gwak, N. Oh, S. I. Yang, S. Kwon, S. Nam, W. I. Park, In-sensor analog optoelectronic processing of concurrent event and memory signals for dynamic vision sensing. *Nat. Commun.* **17**, 1250 (2026).

37. Y. Yang, C. Pan, Y. Li, X. Yangdong, P. Wang, Z.-A. Li, S. Wang, W. Yu, G. Liu, B. Cheng, Z. Di, S.-J. Liang, F. Miao, In-sensor dynamic computing for intelligent machine vision. *Nat. Electron.* **7**, 225–233 (2024).

38. D. Gehrig, D. Scaramuzza, Low-latency automotive vision with event cameras. *Nature* **629**, 1034–1040 (2024).

39. Q.-B. Zhu, B. Li, D.-D. Yang, C. Liu, S. Feng, M.-L. Chen, Y. Sun, Y.-N. Tian, X. Su, X.-M. Wang, S. Qiu, Q.-W. Li, X.-M. Li, H.-B. Zeng, H.-M. Cheng, D.-M. Sun, A flexible ultrasensitive optoelectronic sensor array for neuromorphic vision systems. *Nat. Commun.* **12**, 1798 (2021).

40. Z. Zhang, S. Wang, C. Liu, R. Xie, W. Hu, P. Zhou, All-in-one two-dimensional retinomorphic hardware device for motion detection and recognition. *Nat. Nanotechnol.* **17**, 27–32 (2022).

41. G. Chen, H. Cao, J. Conradt, H. Tang, F. Rohrbein, A. Knoll, Event-based neuromorphic vision for autonomous driving: A paradigm shift for bio-inspired visual sensing and perception. *IEEE Signal Process. Mag.* **37**, 34–49 (2020).

42. C. Cimarelli, J. A. Millan-Romera, H. Voos, J. L. Sanchez-Lopez, Hardware, algorithms, and applications of the neuromorphic vision sensor: A review. *Sensors* **25**, 6208 (2025).

43. P.-Y. Huang, B.-Y. Jiang, H.-J. Chen, J.-Y. Xu, K. Wang, C.-Y. Zhu, X.-Y. Hu, D. Li, L. Zhen, F.-C. Zhou, J.-K. Qin, C.-Y. Xu, Neuro-inspired optical sensor array for high-accuracy static image recognition and dynamic trace extraction. *Nat. Commun.* **14**, 6736 (2023).

44. R. Parada, Neuromorphic solar edge AI for sustainable wildfire detection. *Internet Things* **36**, 101862 (2026).

45. F. Liao, F. Zhou, Y. Chai, Neuromorphic vision sensors: Principle, progress and perspectives. *J. Semicond.* **42**, 013105 (2021).

46. Q. Ren, C. Zhu, S. Ma, Z. Wang, J. Yan, T. Wan, W. Yan, Y. Chai, Optoelectronic devices for in-sensor computing. *Adv. Mater.* **37**, e2407476 (2025).

47. F. Zhou, Z. Zhou, J. Chen, T. H. Choy, J. Wang, N. Zhang, Z. Lin, S. Yu, J. Kang, H.-S. P. Wong, Y. Chai, Optoelectronic resistive random access memory for neuromorphic vision sensors. *Nat. Nanotechnol.* **14**, 776–782 (2019).

48. S. W. Cho, C. Jo, Y.-H. Kim, S. K. Park, Progress of materials and devices for neuromorphic vision sensors. *Nano Micro Lett.* **14**, 203 (2022).
